# Human placental extract ameliorates methotrexate-induced nephrotoxicity in albino rats: ultrastructural, biochemical and biophysical studies

**DOI:** 10.1038/s41598-026-55946-3

**Published:** 2026-06-05

**Authors:** Hoda A. Mahran, Yasmeen M. Gawaan, Mohamed SA. El-Gerbed, Yasser I. Khedr

**Affiliations:** 1 Zoology Department, Faculty of Science, Menoufia University, Shebeen El- Kom, Egypt; 2 Zoology Department, Faculty of Science, Damanhour University, Damanhour, Egypt; 3 Physics Department, Faculty of Science, Damanhour University, Damanhour, Egypt

**Keywords:** Methotrexate, Human placental extract, Nephrotoxicity, Anti-oxidants, Ultrastructure, Rats, Biochemistry, Drug discovery, Medical research, Nephrology, Physiology

## Abstract

Methotrexate (MTX) is a well-known medication for the treatment of different cancer types and autoimmune diseases. The current study target was to measure the capability of human placental extract (HPE) to ameliorate the nephrotoxicity induced by MTX in male albino rats. In the present study, rats were distributed into four groups; a control group (each rat was intraperitoneally injected with 0.5 ml of 0.9% NaCl daily for five days), HPE-treated group (HPE, 10.08 mg/Kg b.w/day, was subcutaneously injected for two weeks), MTX-treated group (MTX, 5 mg/Kg b.w/day, was intraperitoneally injected for five days) and MTX and HPE-treated group (Both MTX and HPE were injected to rats at the same time with the same doses, duration and injection routes in MTX and HPE groups). During the experimental period, clinical observations and body weights of rats were recorded. Rats were dissected after twenty-four hours from the last dose of each group, blood samples were collected for relative blood viscosity measurements and kidneys were also collected for biochemical, ultrastructural and dielectric properties (dielectric constant, dielectric loss and conductivity) investigations. MTX treatment resulted in a highly significant decrease in rat body weights, a highly significant decrease in glutathione (GSH) level and catalase (CAT) activity, a significant decrease in superoxide dismutase (SOD) and glutathione peroxidase (GPx) activities and a highly significant increase in the malondialdehyde (MDA) level and relative blood viscosity compared to the control group. Besides, obvious ultrastructural changes and pronounced decrease in the dielectric properties of kidney tissues were noticed. While HPE treatment with MTX improved body weight, biochemical, ultrastructural and biophysical changes comparing to the MTX group. Human placental extract can reduce MTX-induced nephrotoxicity in rats through boosting oxidative stress/anti-oxidant balance as it is rich with essential elements.

## Introduction

Methotrexate (MTX) has been used as a chemotherapeutic agent with a wide dosing range for a variety of indications^1^. It is an effective drug for the treatment of different cancer types, rheumatoid arthritis, psoriasis, Crohn’s disease and multiple sclerosis treatment^2^. Nephrotoxicity is the term used to describe any renal damage caused by drugs, whether directly or indirectly, including acute renal failure, tubulopathies, and glomerulopathies^3^. Many of the chemotherapeutic drugs, including MTX, are nephrotoxic and can promote kidney dysfunction^4,5^.

Methotrexate raised reactive oxygen species (ROS) levels and inflammatory response in the kidney of rats^5^. It also induced pathological changes, including severe congestion of the renal tissue with marked inflammation, interstitial edema, necrosis and glomerulosclerosis, as well as many ultrastructural changes in the kidney of rats^6^. 

Sahindokuyucu-Kocasari et al.^7^. documented that MTX caused significant elevations in the activities of aminotransferases, levels of creatinine and malondialdehyde (MDA) and a significant decline in the activities of catalase (CAT), glutathione peroxidase (GPx) and superoxide dismutase1 (SOD1) and the level of glutathione (GSH). Moreover, they also noticed that MTX caused histopathological alterations and recorded significant increases in the expression of caspase-3, C-reactive protein, granulocyte colony-stimulating factor and nitric oxide synthase in both liver and kidney tissues of mice. Düğeroğlu and Özgenoğlu^8^ revealed that MTX caused oxidative injury in rats’ kidney and liver tissues. Recently, MTX treatment led to renal damage in rats via elevated induction of oxidative stress, inflammatory cytokines and apoptosis^9^.

The placenta is a discoid organ that allows the physical connection between mother and fetus. The placenta grows to give a wide surface area for materno-fetal exchanges during pregnancy^10^. The placenta is an important organ for women’s health and their fetus during pregnancy. During fetal development, the placenta works as the fetal gastrointestinal, renal, respiratory, immune and endocrine systems^11^.

The history of the placenta as a curing agent is very old. Russian ophthalmologist Filatov^12^ is the first to use the placenta. After many clinical experiments, the author was convinced that some tissues of human or animal origin could be used to obtain a curative effect and named them biogenic stimulators.

The placental extract components depend on the preparation method and the solubility of the components in the respective extraction solvent. Placental extracts are hydroalcoholic and aqueous^13^. Human placental extract (HPE) contains numerous compounds such as cytokines, growth factors^14^, non-essential amino acids^15^, minerals and trace elements^16^.

Human placental extract is used to ameliorate hepatic injury through liver regeneration^17^, to treat chronic hepatitis^18,19^ and to inhibit inflammatory responses and apoptosis^20^. Bak et al.^21^. revealed that HPE had a protective role in hepatocyte apoptosis in vitro and in vivo by suppressing oxidative stress and supporting cell homeostasis. **Bak** et al.^22^. indicated that HPE potentially protects against muscle atrophy and oxidative cell death. Park and Cho^23^ reported that intravenous administration of HPE significantly improved fatigue, pain and health-related quality of life.

Treatment with HPE reduces hepatic fibrosis and early cirrhosis and has anti-inflammatory and anti-oxidant potentials^24^. Recently, Ishikawa et al.^25^. confirmed that HPE acts as a new agent for cirrhosis treatment due to its anti-inflammatory effect on macrophages and senescent cells. This study aimed to evaluate the efficacy of HPE to reduce MTX-induced nephrotoxicity.

## Materials and methods

### Animal selection and care

In the current study, forty adult male albino rats (*Rattus norvegicus*) were obtained from the National Research Centre, Giza, Egypt. They were about 3 months old and weighing about 120 g. These rats were housed in clean cages and were fed a standard diet and water (*ad libitum*). Rats were kept at a temperature of 24 ± 2^ο^C and 12-hour light-dark cycle.

All methods of the current study were carried out in accordance with relevant ethical guidelines and regulations of the ARRIVE guidelines. The ethical approval number of this study is FHI719, and it was obtained from the Faculty of Science, Menoufia University, Egypt.

### Drugs and chemicals

#### Methotrexate

Methotrexate was purchased from Hikma Specialized Pharmaceuticals Company, Egypt, as vials, each one has 50 mg of MTX. It is intraperitoneally injected as it is an effective route for delivering substances in research animals because the peritoneal cavity has a large surface area and is rich with blood supply, allowing for rapid absorption.

#### Human placental extract

The extract of the human placenta was purchased from Japan Bio Products Co. Ltd, Korea, as Laennec ampoules each; 2 ml ampoule contains 112 mg of water HPE that medically can be subcutaneous or intramuscular injected.

### Study design

Rats were randomly distributed into four groups, each group was 10 rats, as follows:

#### Control group

Each rat was intraperitoneally injected with 0.5 ml of 0.9% NaCl daily only for five days.

#### HPE-treated group

HPE (10.08 mg/Kg b.w equivalent to the human therapeutic dose according to Paget and Barnes^26^, was subcutaneously injected to rats daily for two weeks.

#### MTX-treated group

MTX (5 mg/Kg b.w) was intraperitoneally injected to rats for five consecutive days^27^.

#### MTX and HPE-treated group

Rats were injected with MTX (5 mg/Kg b.w) intraperitoneally for five consecutive days and at the same time, HPE (10.08 mg/Kg b.w) subcutaneously daily for two weeks.

In the present study, rats were under observation and their body weights were recorded at the beginning, after five days from the injection and at the end of the experiment (2 weeks).

### Samples collection

After twenty-four hours from the last dose, rats of all groups were physically anesthetized by decapitation by applying pressure at the base of the skull and pulling of the tail to dislocate the neck, and then they were dissected. Blood was withdrawn from the heart to obtain a large volume of blood and it was put in EDTA tubes for relative blood viscosity determination, while kidneys were taken for dielectric properties (dielectric constant, dielectric loss and conductivity) determination, ultrastructural examinations and biochemical analysis.

For dielectric properties measurements, kidneys were immediately used at the frequency range 42 kHz-5 MHz.

For ultrastructural examinations, very small pieces of the kidney cortex were fixed in formalin-glutaraldehyde (_4_F_1_G).

For biochemical analysis, small parts of kidney tissues were preserved at -20℃, and then used for the determination of MDA and GSH levels and CAT, SOD and GPx activities.

### Biochemical analysis

Lipid peroxidation end product (MDA) and anti-oxidant biomarkers (GSH, CAT, SOD & GPx) were assessed by using the Biodiagnostic Company kits in the kidney tissues homogenate. Thiobarbituric acid reacts with MDA in an acidic medium at 95 °C for 30 min to form thiobarbituric acid reactive product. The absorbance of the resultant pink product can be measured at 534 nm by using a colorimetric method according to Ohkawa et al.^28^. While GSH, CAT, SOD & GPx were measured at 405, 510, 560 and 340 nm, respectively, by using colorimetric methods according to Beulter et al.^29^, Aebi^30^, Nishikimi et al.^31^ and Paglia and Valentine^32^, respectively.

### Ultrastructual studies

Small pieces of kidney cortex tissues were fixed in _4_F_1_G for 24 h and processed for ultrastructural studies according to Reynolds^33^. The stained grids were examined and photographed using a Jeol 100 CX transmission electron microscope at the Faculty of Science, Alexandria University, Alexandria, Egypt.

### Biophysical studies

#### Relative blood viscosity

Relative blood viscosity was measured using low-cost syringe. A whole blood sample (2 ml) was put in a syringe, then it was set in a vertical position, and the syringe plunger was removed. The blood was allowed to flow freely, and the blood flow time was recorded by using a stopwatch^34^. The relative blood viscosity was calculated by

Relative blood viscosity = t blood / t water.

t blood is the whole blood (2 ml) flow time, and t water is the distilled water (2 ml) flow time, which was used as a standard.

#### Dielectric properties study of the kidney tissues

The measurement of the dielectric properties of the kidney tissues were carried out in 42 kHz-5 MHz frequency range^35^ using a Loss Factor Meter type HIOKI 3532 LCR Hi TESTER, version 1.02, Japan and cell types (PW 950/60) manufactured by Philips, Faculty of Science, Damanhour University, Damanhour, Egypt.

The dielectric constant (έ) of the sample was calculated for each frequency using the following equation:

$${\rm \acute{\varepsilon} = \frac{cd}{\varepsilon_0 A}}$$

A is the electrode area, d is the distance between the two electrodes and ε_0_ is the permittivity of free space.

The dielectric loss (ε”) was calculated from the following equation:

$$\varepsilon'' = \frac{\acute{\varepsilon}}{2\pi fRC}$$

f is the applied frequency in Hertz, R and C are the resistance and capacitance of the sample at resonance.

The electric conductivity (σ) was calculated from the following equation:

$$\sigma = 2\pi f \varepsilon'' \varepsilon_0$$

### Statistical analysis

The statistical analysis of the present study was done by the Statistical Package for the Social Science (SPSS) program, version 20, using a one-way ANOVA test. The obtained data are expressed as mean ± SE, and the statistical significance between groups was estimated by the least significant difference post hoc test, as *p* ≤ 0.05, *p* ≤ 0.01 and *p* ≤ 0.001^36^.

## Results

### Changes in rats’ body weights

**Fig. 1:**
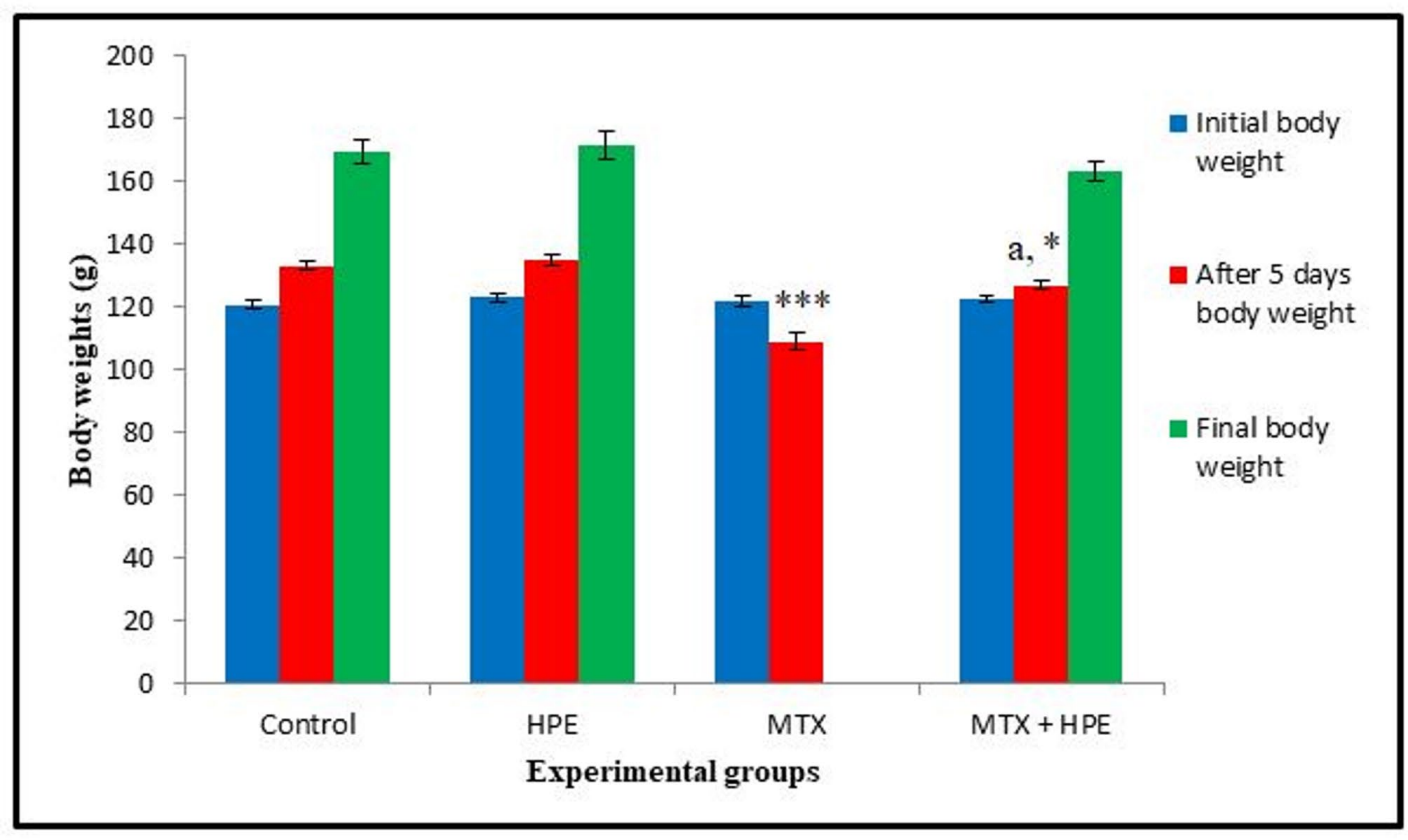
Body weight changes of rats of the different experimental groups. n₌ 10. *** highly significant at *p* ≤ 0.001 & *significant at *p* ≤ 0.05 versus the control group. ^**a**^ highly significant at *p* ≤ 0.001 versus MTX-treated group.

The body weights; initial, after 5 days of injection and the final, of rats of all the studied groups are represented in Fig. 1. The initial body weights of rats recorded an insignificant difference (*p* > 0.05) between the studied groups.

Rats treated with HPE recorded insignificant increases (*p* > 0.05) in their body weights (after 5 days and final body weight) compared with the control rats.

After 5 days of MTX injection, there was a highly significant reduction (*p* ≤ 0.001) in the body weights of rats as compared with the control animals at the same period. Moreover, the body weights of MTX and HPE-treated group recorded a highly significant increase (*p* ≤ 0.001) at the fifth day compared with MTX-treated animals within the same period (5 days), but it was still showing a significant decrease at *p* ≤ 0.05 compared with the control animals.

The final body weights of MTX and HPE-treated group recorded an insignificant decrease (*p* > 0.05) compared with the control group.

### Lipid peroxidation and anti-oxidant biomarkers in kidney tissues

#### Malondialdehyde

**Fig. 2:**
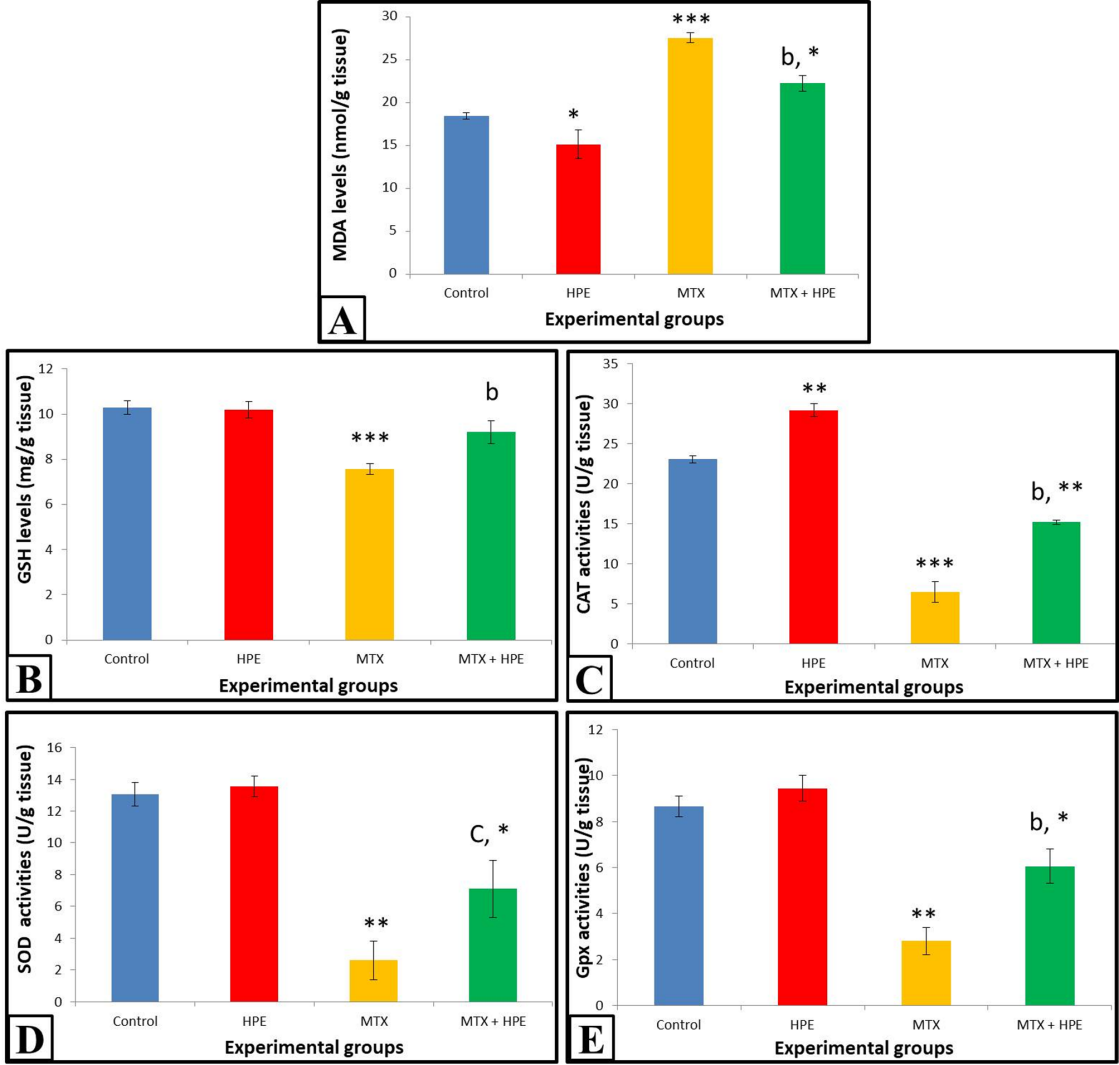
**(A)** MDA levels, **(B)** GSH levels, **(C)** CAT activities, **(D)** SOD activities and **(E)** GPx activities in the kidney tissues of animals of the different experimental groups. n₌ 5. ***highly significant at *p* ≤ 0.001, **significant at *p* ≤ 0.01 and *significant at *p* ≤ 0.05 versus the control group. ^b^ significant at *p* ≤ 0.01 and ^c^ significant at *p* ≤ 0.05 versus MTX-treated group.

Data in Fig. 2A represented tissue MDA levels in animals of the different studied groups. Human placental extract-treated rats showed a significant decrease (*p* ≤ 0.05) in MDA level as compared with the control group. On the other hand, a highly significant increase (*p* ≤ 0.001) was recorded in MDA level of MTX-treated rats compared with the control group.

A significant decrease (*p* ≤ 0.01) was recorded in MDA level in MTX and HPE-treated group, compared with the MTX group, although this group was still recording a significant increase (*p* ≤ 0.05) when compared with the control group.

#### Anti-oxidant biomarkers

Tissue GSH levels and CAT, SOD and GPx activities of the studied groups are shown in Fig. 2(B-E).

Rats treated with HPE recorded insignificant differences (*p* > 0.05) in GSH levels and SOD and GPx activities, while they recorded a significant increase (*p* ≤ 0.01) in CAT activity compared with the control group.

Methotrexate-treated rats recorded highly significant decreases at *p* ≤ 0.001 in GSH level and CAT activity and significant decreases at *p* ≤ 0.01in SOD and GPx activities when compared with the control group.

MTX and HPE-treated rats recorded significant increases at *p* ≤ 0.01 in GSH level and CAT and GPx activities and at *p* ≤ 0.05 in SOD activity compared with MTX-treated rats. On the other hand, compared with the control rats, MTX and HPE-treated rats showed an insignificant decrease (*p* > 0.05) in GSH level, while significant decreases at *p* ≤ 0.01 in CAT activity and at *p* ≤ 0.05 in SOD and GPx activities were still recorded.

### Ultrastructural observations

#### Control group

**Fig. 3:**
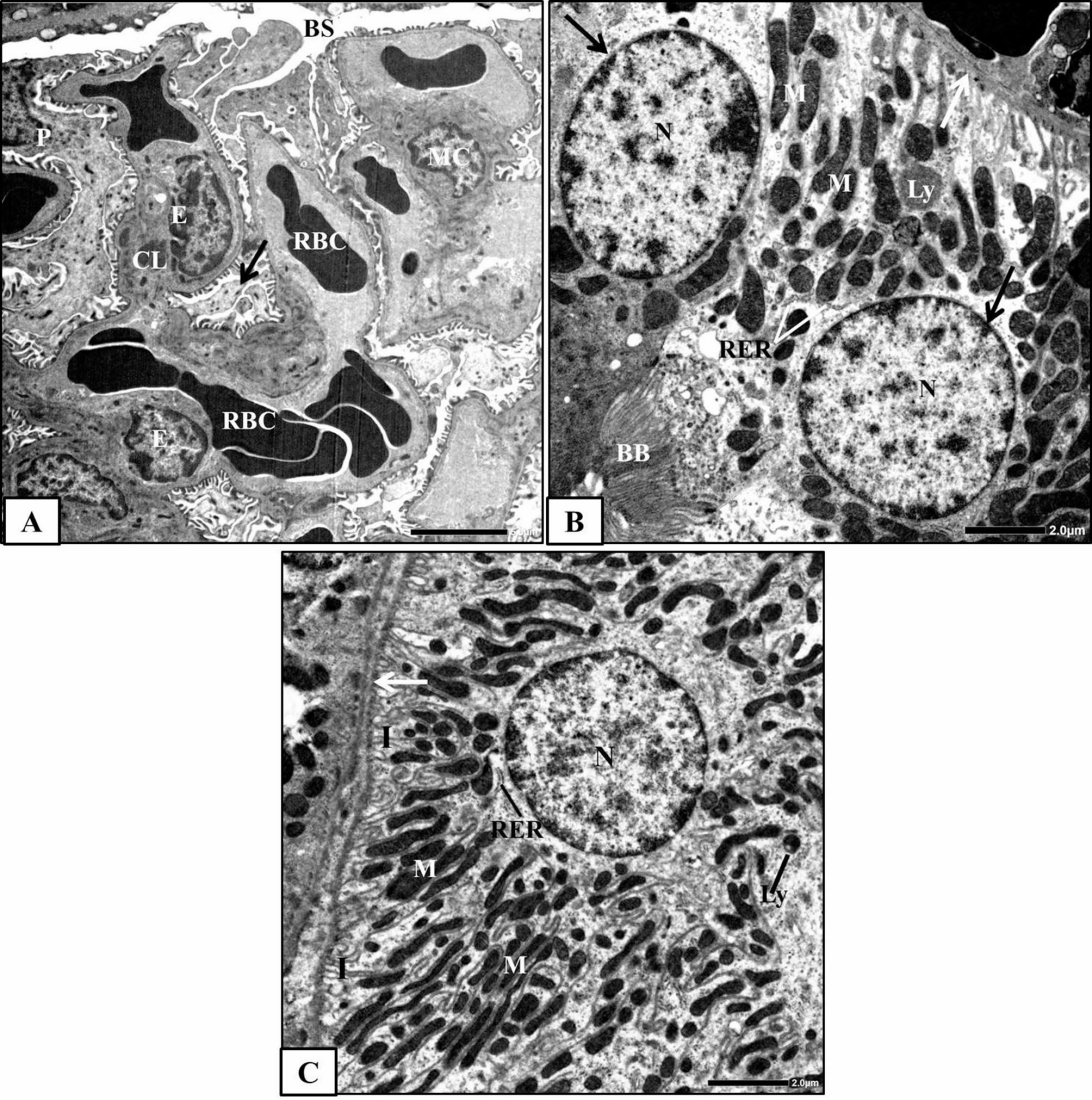
Electron micrographs obtained from kidney cortex of the control rats **(A)** a part of normal Malpighian corpuscle showing glomerular capillary lumen (CL), red blood cells (RBC), endothelial cells (E), podocyte (P), foot processes (arrow), mesangial cell (MC) and Bowman’s space (BS), **(B)** a part of normal proximal convoluted tubule showing lining cell with basement membrane (white arrow), nuclei (N) with regular nuclear envelope (black arrows), numerous mitochondria (M), lysosome (Ly), rough endoplasmic reticulum (RER) and brush border (BB) and **(C)** a part of normal distal convoluted tubular cell showing basement membrane (white arrow), spherical nucleus (N), lysosome (Ly), mitochondria (M) between the infoldings (I) and rough endoplasmic reticulum (RER).

The control rat’s renal cortex ultrastructural examination showed normal architecture of Malpighian corpuscles and renal tubules (proximal and distal convoluted tubules). The Malpighian corpuscle consists of Bowman’s capsule encloses the corpuscle, a capillary tuft (glomerulus) and Bowman’s space between them. Bowman’s capsule has two faces; the outer face, known as the parietal face that comprises simple squamous epithelium, while the inner face, known as the visceral face that comprises a specialized layer of modified cells, podocytes, overlying the glomerular capillaries. The glomerulus consists of a network of capillary loops made up from a thin layer of fenestrated endothelium, a central region of mesangial cells with surrounding matrix and the visceral epithelial cells (podocytes) overlying the capillaries. The podocytes are the largest cells in the glomerulus, have many primary processes which terminate in numerous secondary processes surrounding the capillaries, and finally are divided into foot processes. The foot processes connect the podocyte body with the glomerular basement membrane (Fig. 3A). The glomerular capillary wall or filtrating barrier consists of three layers; the fenestrated capillary endothelium, the glomerular basement membrane and the filtration slit diaphragm between the foot processes of the visceral epithelial cells.

Proximal convoluted tubules are lined with simple cuboidal epithelial cells based on a well-defined basement membrane. The lining cells contain large rounded central nucleus surrounded by a regular envelope. The apical surfaces of these cells have brush borders that consist of numerous long regularly oriented, and closely packed thin finger-like projections, microvilli, which project into a narrow tubular lumen. The lining cells are characterized by infoldings from their basal plasma membrane and numerous elongated mitochondria that lie longitudinally intervening between the infoldings. Thin and line-shaped rough endoplasmic reticulum, numerous lysosomes (Fig. 3B) and numerous apical vesicles were also observed.

The distal convoluted tubules are lined with short and flat cuboidal epithelial cells that don’t have brush borders. These cells are characterized by apical round nucleus, extensive basal plasma membrane infoldings, elongated mitochondria between them and rough endoplasmic reticulum (Fig. 3C). Few lysosomes were also noticed in their cytoplasm.

#### Human placental extract-treated group

**Fig. 4:**
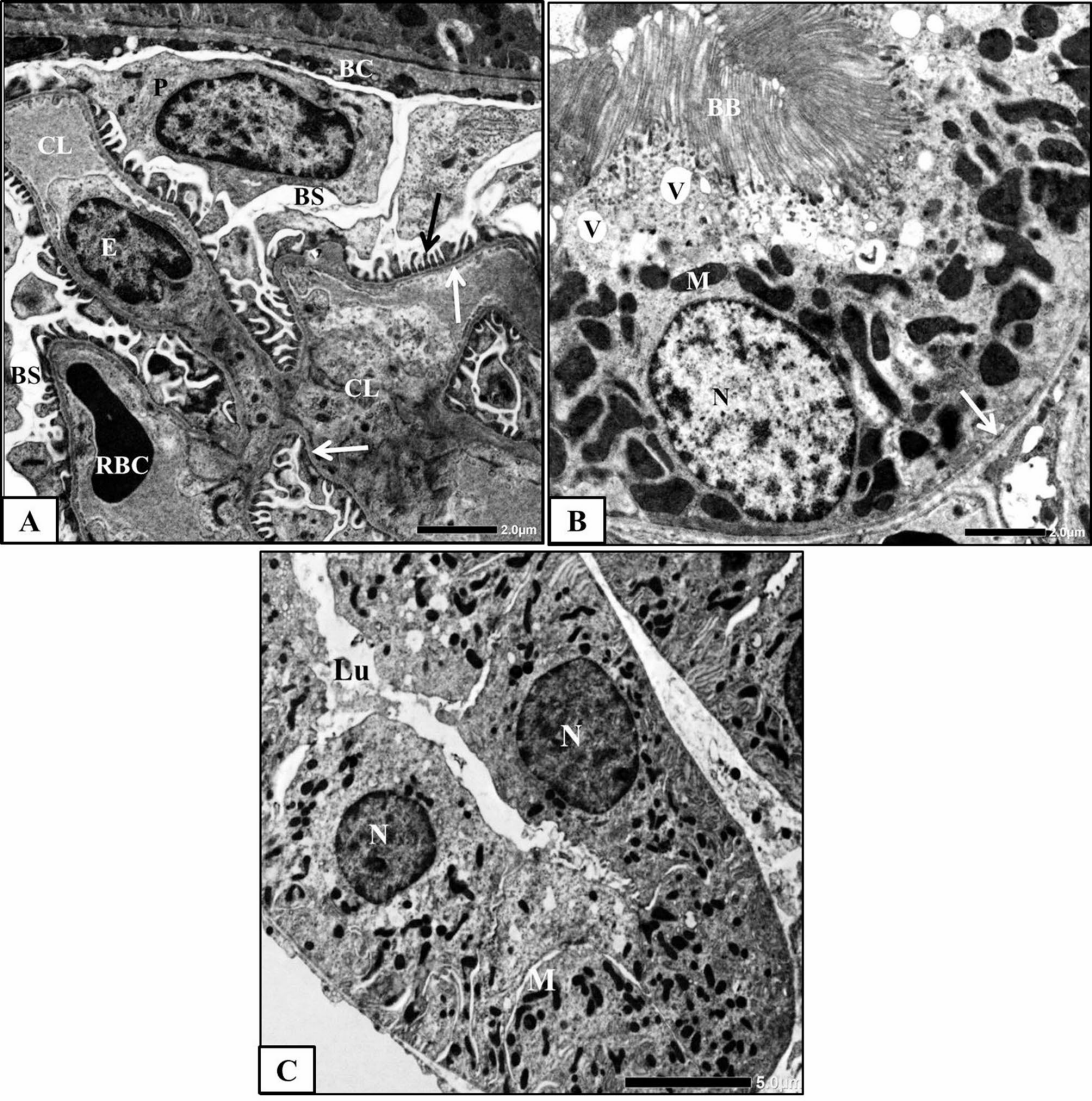
Electron micrographs obtained from renal cortex of HPE-treated rats; **(A)** a part of normal Malpighian corpuscle showing Bowman’s capsule (BC), Bowman’s spaces (BS), glomerular capillary lumens (CL), endothelial cell (E), glomerular basement membrane (white arrows), foot processes (black arrow), red blood cell (RBC) and podocyte (P), **(B)** normal proximal convoluted tubular cell with basement membrane (white arrow), nucleus (N), mitochondria (M), brush border (BB) and apical vesicles (V) and **(C)** normal distal convoluted tubule with lumen (Lu) and normally arranged tubular cells with round nuclei (N) and mitochondria (M).

The ultrastructural examination of the renal cortex of HPE-treated rats revealed high similarity with that of the control rats. Normal glomerular ultrastructure was observed (Fig. 4A). Proximal and distal convoluted tubules maintained their typical ultrastructure. The tubular cells appeared with distinct cellular margins and rested on a well-defined basement membrane. The mitochondria were normally observed and distributed within the cytoplasm (Fig. 4B&C). The brush borders of the proximal convoluted tubular cells appeared with an organized shape and orientation (Fig. 4B).

#### Methotrexate-treated group

The electron microscopic examination of the renal cortex of MTX-treated rats showed extensive alterations and abnormalities.

**Fig. 5:**
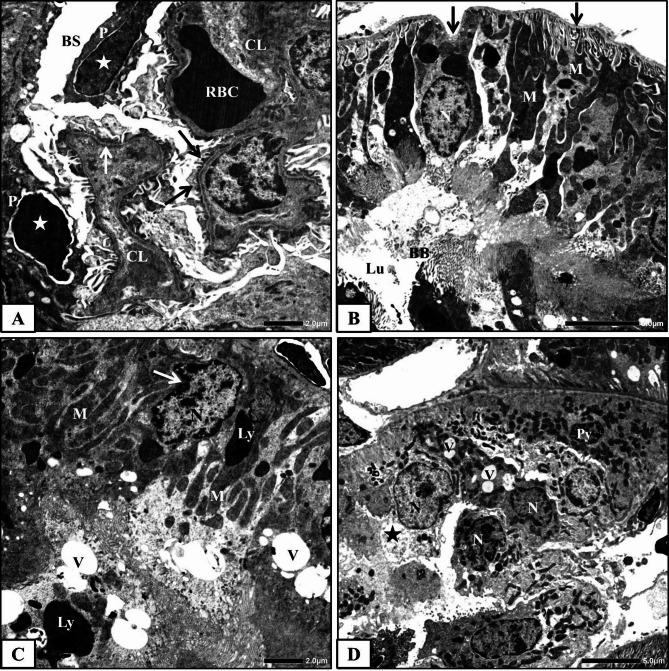
Electron micrographs obtained from kidney tissues of MTX-treated rats showing **A)** a part of glomerulus from showing capillary lumens (CL), thickened glomerular basement membrane (white arrow), red blood cell (RBC), degenerated podocytes (P) with dark nuclei (stars), disorganized and deformed foot processes (black arrows) and widen Bowman’s space (BS), **B&C)** proximal convoluted tubules showing irregular basement membrane (black arrows), irregular nucleus (N) with nuclear pocket (white arrow), degenerated mitochondria (M), heterogenic secondary lysosomes (Ly), numerous apical vacuoles (V), fragmented brush border (BB) and dilated lumen (Lu) and **D)** a part of a severely affected distal convoluted tubule with disorganized and degenerated lining cells, lyzed cytoplasmic material (star), pyknotic nucleus (Py), irregular nuclei (N) and vacuoles (V).

Malpighian corpuscles and Bowman’s capsules appeared with degenerated parietal cells contained dark condensed nuclei, while Bowman’s spaces were dilated and glomerular capillaries were distorted. Some of the mesangial cells were highly deteriorated. The three layers of the filtrating barrier were damaged, sometimes with thickened glomerular basement membrane, degenerated fenestrated endothelial lining and deformed or fragmented foot processes. Moreover, degenerated podocytes with dark nuclei were observed (Fig. 5A).

Proximal convoluted tubules showed severe abnormalities in their architecture. Their lining cells showed pleomorphic and indistinct cellular margins (Fig. 5B). The cytoplasmic material of these cells was degenerated. A Large number of apical vacuoles and numerous heterogenic secondary lysosomes were commonly seen. Most of the mitochondria were swollen or degenerated (Fig. 5B&C). Some nuclei appeared pyknotic with condensed chromatin materials, while others were irregular with nuclear pockets (Fig. 5C). The microvilli were fragmented, disoriented and disappeared in certain areas, resulting in wider tubular lumens (Fig. 5B).

Most of the distal convoluted tubular cells were degenerated and disorganized. The cytoplasmic material of some cells was lysed, with degenerated mitochondria and numerous vacuoles, while the nuclei appeared pyknotic or with irregular nuclear envelopes, sometimes forming nuclear pockets (Fig. 5D).

#### Methotrexate and HPE-treated group

**Fig. 6:**
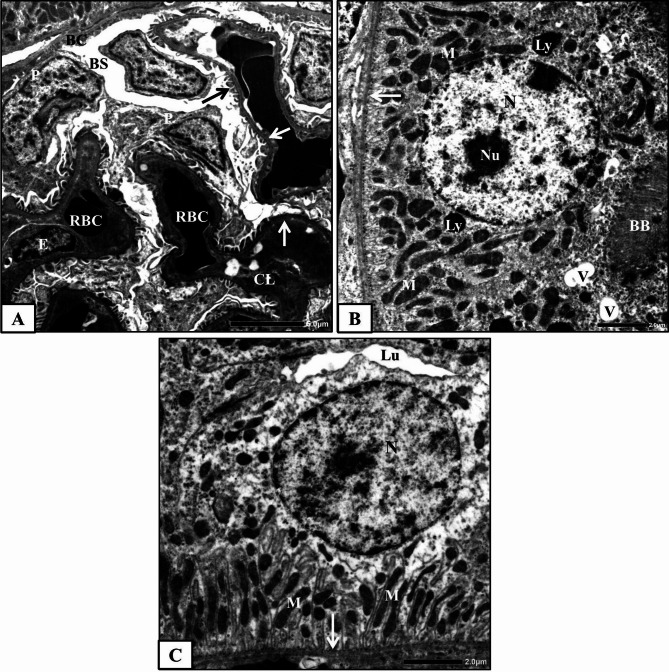
Electron micrographs of obtained from kidney tissues of MTX and HPE-treated rats showing **(A)** a part of nearly normal Malpighian corpuscle showing Bowman’s capsule (BC), Bowman’s space (BS), glomerular capillary lumen (CL), endothelial cell (E), glomerular basement membrane (white arrows), foot processes (black arrow), podocytes (P) and red blood cells (RBC), **(B)** a part of nearly normal proximal convoluted tubular cell showing basement membrane (arrow), round nucleus (N), nucleolus (Nu), mitochondria (M), lysosomes (Ly), few apical vacuoles (V) and brush border (BB) and **(C)** a part of distal convoluted tubule showing normal lumen (Lu) and nearly normal lining epithelial cell with normal basement membrane (arrow), round nucleus (N) with regular envelope and mitochondria (M).

Electron microscopic examination of the renal cortex of MTX and HPE-treated rats showed an apparent degree of amelioration compared with MTX-treated rats. In these specimens, most of the Malpighian corpuscles restored nearly the normal ultrastructure of their parts; glomerular capillary network, podocytes, mesangial cells and filtration barrier (Fig. 6A).

The majority of the proximal convoluted tubular cells appeared healthy with definite basement membranes and well-developed brush borders. The majority of mitochondria were normal and uniformly arranged. Furthermore, the cytoplasm appeared homogenous and contained few lysosomes and vacuoles (Fig. 6B).

Most of the distal convoluted tubules appeared nearly normal with normal lumens and lining cells. The lining epithelial cells appeared with normal basement membranes and contained normal nuclei and mitochondria (Fig. 6C). Lysosomes as well as vacuoles were less abundant than MTX group.

### Biophysical results

#### Relative blood viscosity

**Fig. 7:**
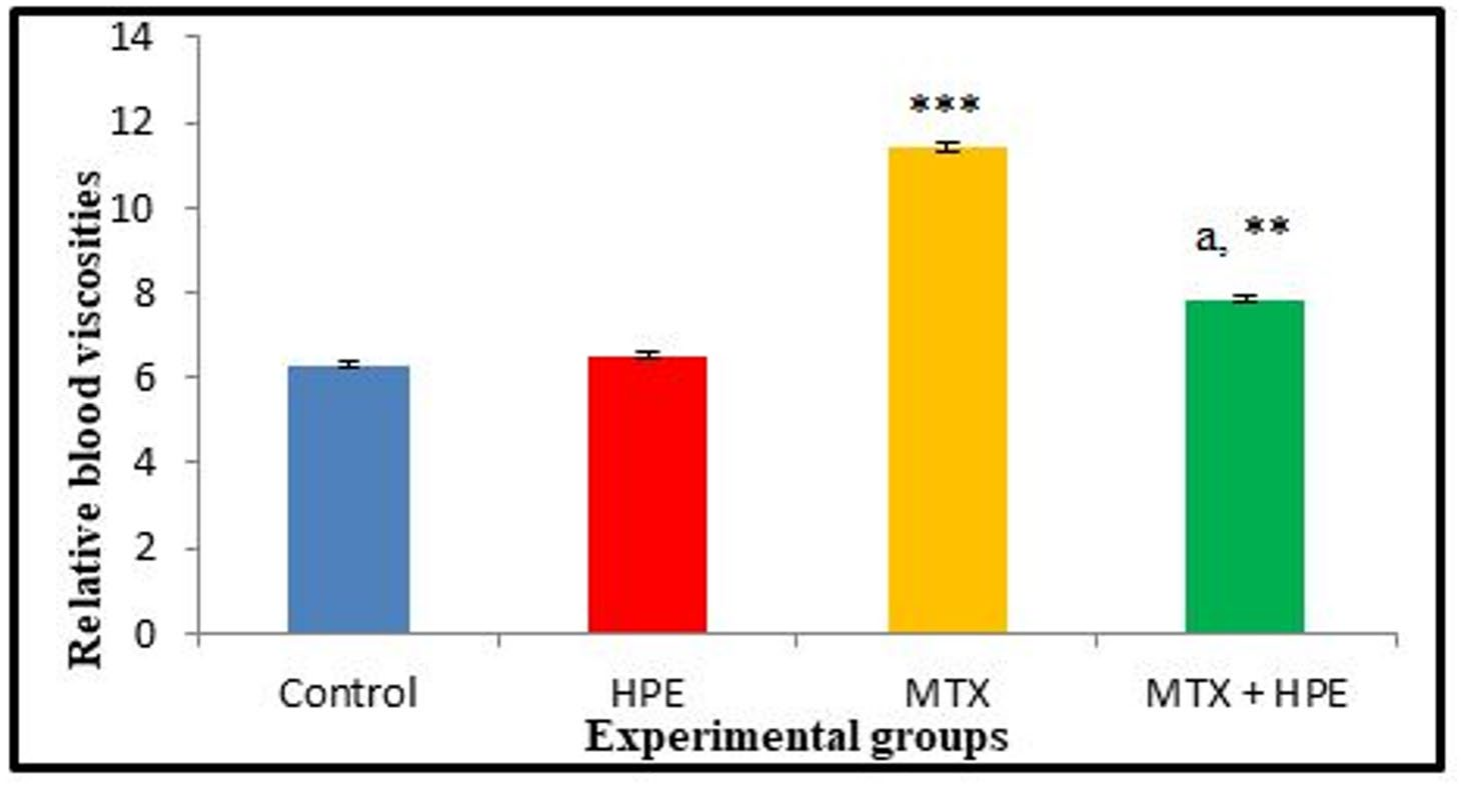
The relative blood viscosity of animals of the different experimental groups. n₌ 5. *** highly significant at *p* ≤ 0.001 and **significant at *p* ≤ 0.01 versus the control group. ^a^ highly significant at *p* ≤ 0.001 versus MTX-treated group.

Data in Fig. 7 represented the relative blood viscosity of all the experimental rats. There was an insignificant increase (*p* > 0.05) in the relative blood viscosity between the control rats and HPE-treated rats. On the other side, MTX-treated rats showed a highly significant increase at *p* ≤ 0.001 in their relative blood viscosity compared with the control rats.

Relative blood viscosity of MTX and HPE-treated rats recorded a highly significant decrease (*p* ≤ 0.001) compared with MTX-treated rats, although it was still recording a significant increase (*p* ≤ 0.01) compared with the control rats.

#### Dielectric properties of the kidney

The dielectric relaxation spectroscopy showed a dielectric dispersion in the frequency range 42 kHz-5 MHz for all the experimental groups.

**Fig. 8:**
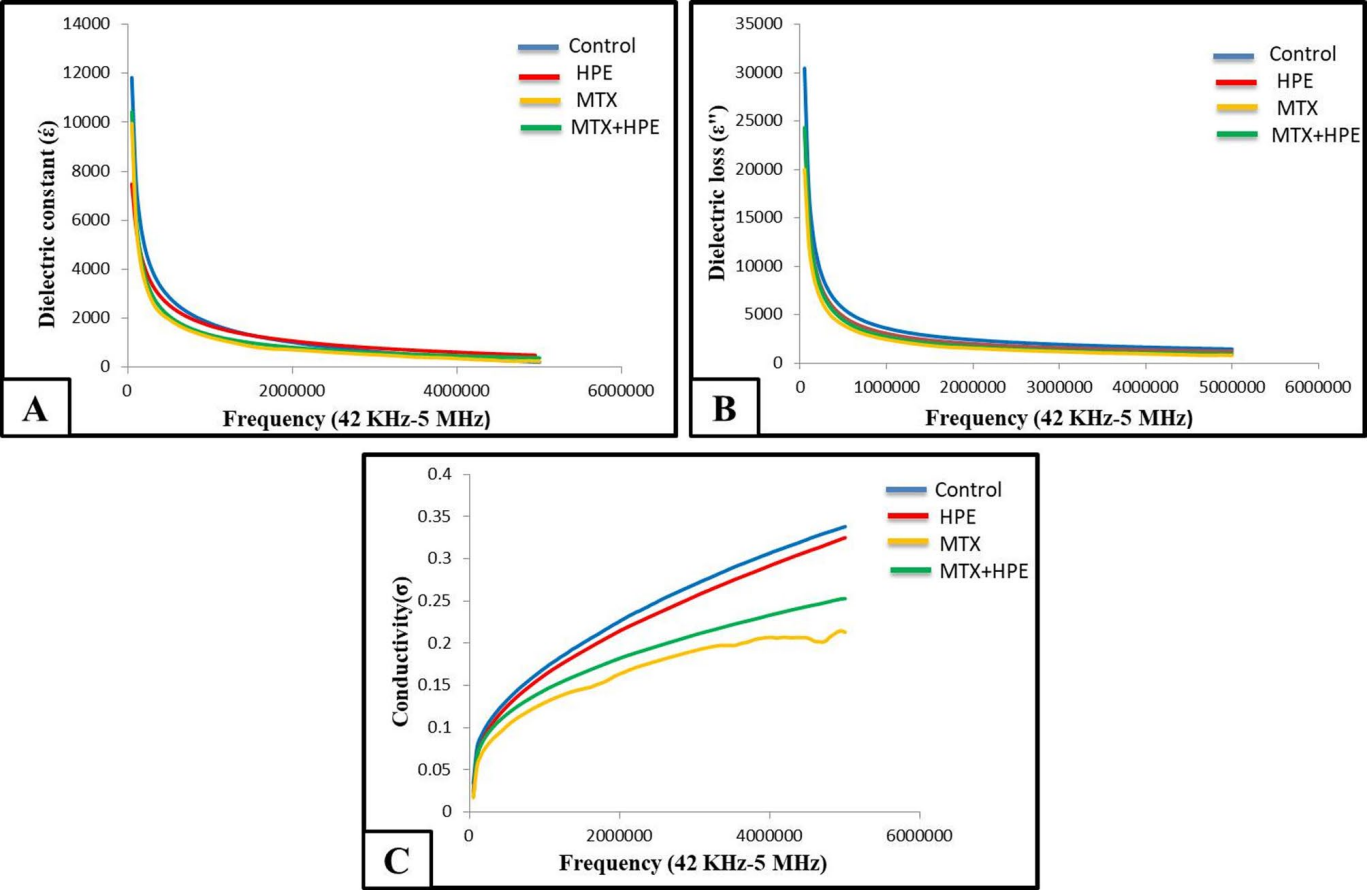
**A)** Variation of the dielectric constant (έ), **B)** variation of the dielectric loss (ε˝) and **C)** variation of the conductivity (σ) of the kidney tissues of all the experimental groups.

Kidney tissues of HPE-treated rats didn’t show difference in the dielectric properties; dielectric constant (Fig. 8A), dielectric loss (Fig. 8B) and conductivity (Fig. 8C), comparing with the control group.

Kidney tissues of MTX-treated rats showed a pronounced decline in the dielectric constant (Fig. 8A), dielectric loss (Fig. 8B) and conductivity (Fig. 8C) comparing with the control group. Kidney tissues of MTX and HPE-treated rats recorded an increase in the dielectric constant (Fig. 8A), dielectric loss (Fig. 8B) and conductivity (Fig. 8C) as compared with MTX-treated rats, but the three properties were still showing a decrease compared with the control group.

## Discussion

Acute kidney injury may result from drugs through the induction of tubulointerstitial inflammation, acute interstitial nephritis that results from T cells-mediated immune response stimulation and drugs insolubility in the urine, causing their precipitation as crystals within distal tubular lumens. It can also result from intratubular obstruction, direct tubular injury and localized inflammation^37^.

In the present work, rats treated with MTX recorded a highly significant decline in their body weights, which may be due to loss of appetite. These observations were in harmony with David et al.^38^, who documented that MTX caused body weight loss in rats, and they attributed it to MTX direct toxicity and reduced food and water intake.

In the current study, MTX treatment induced a highly significant elevation in MDA level, a highly significant decline in GSH level and CAT activity and a significant decrease in SOD and GPx activities compared with the control rats. These results may be owing to MTX induced-oxidative stress, which leads to oxidant/anti-oxidant imbalance. Under normal condition, the organism is equipped with efficient defense against oxidative stress, based on the anti-oxidants mainly SOD, CAT and GPx. Both increased production of ROS and lowered anti-oxidant defense, resulting in impaired oxidant/anti-oxidant balance^39^.

Mahmoud^40^ reported that MTX was able to disturb the oxidant/anti-oxidant balance as indicated by increasing MDA level, decreasing GSH level and both SOD and CAT activities in the liver of rats. Depletion in GSH level, after MTX treatment, led to a defective anti-oxidant response and consequently impairment of the cell integrity^41^. 

Heidari et al.^42^. attributed the toxic effect of MTX to mitochondrial dysfunction and oxidative stress through interaction with molecular oxygen, triggering a series of reactions that release reactive oxygen and nitrogen species. These changes lead to a reduction in tissue anti-oxidants, which in turn disrupts the balance between oxidants and anti-oxidants, ultimately causing tissue damage.

Similarly, Adikwu and Bokolo^43^ recorded a significant rise in MDA level and a significant decline in GSH level and SOD, CAT and GPx activities in the kidney tissue of MTX-treated rats, and they attributed these results to free radicals production, which might be the probable reason for the reduction of the anti-oxidant activities. Abouelela et al.^44^. and Jafaripour et al.^45^. reported that MTX treatment strongly disturbed the balance between oxidant and anti-oxidant in the kidney tissue of rats, evidenced by a notable rise in MDA levels, a considerable reduction in GSH levels and SOD and CAT activities.

In the current study, several ultrastructural alterations were noticed in the kidney tissue of MTX-treated rats. These changes included distorted glomeruli, dilated Bowman’s spaces, degenerated podocytes with fragmented foot processes and degenerated tubular epithelial cells with cytoplasmic vacuolization, pyknotic nuclei, degenerated mitochondria and numerous lysosomes. In addition, fragmented microvilli were also seen. These alterations might be related to MTX-induced oxidative stress or its direct toxicity. Çaglar et al.^46^. reported that the main causal factors related to MTX-induced renal injury are ROS. Elsawy et al.^47^. attributed the ultrastructural changes induced by MTX in rats to its precipitation and subsequent direct toxic effects on the renal tubules. 

Pavenstädt et al.^48^. reported that foot processes of podocytes play a major role in the selective permeability of glomeruli, so podocytes injury leads to foot processes retraction then proteinuria, a hallmark of most glomerular diseases. 

The damaged brush borders that were noticed in the present study may be due to the toxicity of MTX or its metabolites. Damage of the brush borders could have been a result of toxin binding to the brush borders membrane^49^. The proximal tubular epithelial cells microvilli significantly expand the luminal surface area, enhancing the tubular reabsorption of vital nutrients therefore, any damage to them can severely impact the kidney’s filtration, absorption, and secretion functions^50^.

The mitochondrial damage that was noticed in the present work may be due to the oxidative stress induced by MTX toxicity. Elbakary et al.^51^. reported that the generation of ROS causes damage to the mitochondria by creating a channel known as the mitochondrial permeability transition pore. The opening of this channel decreases the mitochondrial membrane potential and alters pH levels, leading to a failure in oxidative phosphorylation and a gradual depletion of adenosine triphosphate. Mitochondria contain various proteins that can trigger apoptotic pathways. Damage to the mitochondrial membrane can increase its permeability, resulting in the release of these proteins into the cytosol, ultimately leading to cell death via apoptosis.

The numerous lysosomes that were noticed in the present work may be related to autophagy stimulation by MTX toxicity. Acute pathological stimuli, like the toxic side effects of medications and urinary tract blockages, enhance autophagy in kidney tubules, helping to protect the kidneys from tubular injury and the subsequent fibrosis^52^. Cui et al.^53^. reported that autophagy, as a cellular stress response that is crucial to the pathogenesis, is stimulated in proximal tubules in acute kidney injury.

 Çaglar et al.^46^. observed similar ultrastructural changes in the rat’s kidney after MTX treatment, and they attributed these alterations to the direct toxic effects of MTX metabolites that affect membrane permeability. Wani et al.^54^. observed nearly similar results of our ultrastructural observations in MTX-treated rats and reported that rats administered a low dose of MTX (1 mg/kg once weekly) had severe renal tissue damage and disturbance in renal functions, which was linked to elevated oxidative stress as well as MTX’s pro-inflammatory and pro-apoptotic effects.

In the current study, MTX induced a highly significant increase in the relative blood viscosity compared with the control rats, which may be attributed to the dehydration and hypernatremia that were observed after MTX treatment in our previous study^55^. Increased relative blood viscosity is associated with abnormal renal function^56^. Blood viscosity could be a major factor that can change the evolution of renal impairment^57^. Dehydration causes mild hypernatremia that gradually leads to high blood viscosity, hemoconcentration, inflammatory signals, platelet activation and aggregation, endothelial cells adhesiveness and thrombogenesis^58^.

In the current study, MTX caused a pronounced decline in the dielectric constant, dielectric loss and the conductivity of kidney tissue, which may be attributed to MTX-induced kidney tissue damage. The changes in the polarization properties of the damaged tissue cause a decrease in the dielectric constant value^59^.

In the current study, HPE treatment with MTX resulted in a relative amelioration in body weight changes, biochemical, ultrastructural and biophysical alterations when compared with the MTX group. The improved clinical symptoms and body weight may be due to the bioactive molecules content of HPE. Human placental extract contains a variety of biomolecules that may treat various illnesses and improve the general health of both humans and animals, such as growth factors, cytokines, neurotransmitters, amino acids, enzymes, hormones, vitamins and minerals^60^. Yamauchi et al.^61^. reported that HPE led to suppression of the body weight reduction through its ability to prevent atrophy of adipose tissue and skeletal muscle and lowering oxidative cell death.

In the present study, HPE significantly lowered the elevated MDA level and increased CAT, SOD and GPx activities and GSH level in MTX-treated rats. This improvement may be attributed to anti-oxidant contents and free radical scavenging capacity of HPE. Owing to HPE content of collagen peptides, uracil, tyrosine, phenylalanine and tryptophan, which give it its anti-oxidant properties, it can be used in illnesses linked to oxidative stress treatment^62^. In different in vitro models, HPE gave protection by suppressing lipid peroxidation, nitric oxide, hydroxyl radical and superoxide anion generation^63^.

Our results were in accordance with Shinde et al.^64^. who documented that HPE significantly reduced MDA level and increased SOD, CAT and GPX activities in the brain and liver of mice due to its free radicals scavenging activity and its stimulation of metabolism of oxidative wastes like lipid peroxides. In addition, Lee et al.^65^. reported that HPE plays a role in oxidative stress elimination and anti-oxidant enzymes activation due to its amino acids content.

In the present work, HPE ameliorated the ultrastructural alterations in the rat’s renal cortex resulted from MTX treatment, which may be a result of HPE anti-oxidant, anti-inflammatory and cellular regeneration ability. These observations were linked and parallel to the histological observations in our previous study^55^. Mitchell et al.^66^. revealed that the placental extracts are easily absorbed by attaching to particular receptors on the surface of the targeted cells, which then stimulate the body’s damaged or inactive cells, tissues, and organs to promote tissue regeneration and repair. The ability of human placental extract to promote tissue regeneration and cell proliferation may be explained by the presence of growth factors, hormones, proteins, glycosaminoglycans, nucleic acids, polydeoxyribonucleotides, antibodies, and other nutrients^62^. Furthermore, liver regeneration was associated with HPE content of cytokines and growth factors according to Lee et al.^65^.

In the current study, a highly significant decrease in the relative blood viscosity and pronounced elevation in the dielectric constant, dielectric loss and conductivity of kidney tissue were recorded in rats treated with MTX and HPE compared with the MTX rats. These results may be attributed to HPE treatment that has the capacity to ameliorate kidney functions and enhance cellular regeneration.

## Conclusions

It is clear that HPE, due to its richness with therapeutic components, improved the biochemical parameters and enhanced most of the ultrastructural and biophysical alterations resulted from MTX treatment.

### Recommendations

Further investigations are recommended to carry out on the possible ameliorative effects of HPE to minimize the side effects of drugs on the different mammalian organs.

## Data Availability

The datasets of the current study are available with the corresponding author on reasonable request.
